# Guidance on how to involve people with lived experience in research on digital mental health interventions

**DOI:** 10.1192/j.eurpsy.2024.171

**Published:** 2024-08-27

**Authors:** I. Wells, E. Thelwell, D. Giacco

**Affiliations:** University of Warwick, Coventry, United Kingdom

## Abstract

**Introduction:**

Digital technologies and interventions (defined as patient-facing or self-administered interventions delivered through a digital platform) have an increasing role in mental health care. It is key to ensure that appropriate patient and public involvement (PPI) is not overlooked when developing new digital mental health interventions (DMHIs). The unique perspective offered by those with lived experience can improve study conduct and design as well as ensure that interventions meet the needs of users, which may improve their quality and acceptability.

**Objectives:**

To develop guidance for the involvement of people with lived experience of severe mental illness in designing and conducting research on DMHIs.

**Methods:**

Four co-production workshops were arranged online with people who have lived experience of severe mental illness. Initial ideas were formulated in the first workshop and were then prioritised in the second workshop using the nominal group technique. The prioritised ideas were then refined in workshops three and four. Minutes were generated from each workshop and were validated by the those who took part. These minutes were analysed using thematic analysis.

**Results:**

Nineteen people with lived experience participated in the co-production workshops overall. Six people took part in one workshop only and 13 took part in multiple workshops. Two main themes were identified in this study: why do people become and remain involved in PPI?; and what areas should be discussed within PPI consultations? Three subthemes associated with the second theme were also identified. These are: areas related to specific types of DMHI; areas related to any type of DMHI; and what can make a difference within DMHIs? To ensure that people become and remain interested in PPI around DMHIs, it is important to provide a non-judgemental space for people with lived experience to discuss any concerns and ensure they feel valued during consultations. Aspects to consider discussing in PPI consultations around the development of DMHIs include the provision of safety and security within DMHIs, issues around digital exclusion and the potential impact of people’s symptoms on DMHI use. Finally, points that were identified as important to consider when developing DMHIs include offering encouragement throughout the DMHI, accommodating for individual and collective needs within DMHIs and developing a structure within the DMHI which includes adding in attainable goals.

**Conclusions:**

If used, the information provided from this study can generate positive and productive PPI consultations where those with lived experience can make significant contributions to the development of DMHIs. Such contributions will increase the acceptability and efficacy of the DMHIs developed.

**Disclosure of Interest:**

None Declared

